# Addressing the First Delay in Saving Mothers, Giving Life Districts in Uganda and Zambia: Approaches and Results for Increasing Demand for Facility Delivery Services

**DOI:** 10.9745/GHSP-D-18-00343

**Published:** 2019-03-11

**Authors:** Florina Serbanescu, Mary M. Goodwin, Susanna Binzen, Diane Morof, Alice R. Asiimwe, Laura Kelly, Christina Wakefield, Brenda Picho, Jessica Healey, Agnes Nalutaaya, Leoda Hamomba, Vincent Kamara, Gregory Opio, Frank Kaharuza, Curtis Blanton, Fredrick Luwaga, Mona Steffen, Claudia Morrissey Conlon

**Affiliations:** a Division of Reproductive Health, U.S. Centers for Disease Control and Prevention, Atlanta, GA, USA.; b U.S. Public Health Service Commissioned Corps, Rockville, MD, USA.; c Baylor College of Medicine Children's Foundation-Uganda, Kampala, Uganda.; d Division of Reproductive Health, U.S. Centers for Disease Control and Prevention, Atlanta, GA, USA. Now with Deloitte Consulting, LLP, Atlanta, GA, USA.; e Social and Behavior Change, The Manoff Group, Washington, DC, USA.; f Infectious Diseases Institute, College of Health Sciences, Makerere University, Kampala, Uganda.; g U.S. Agency for International Development, Lusaka, Zambia. Now based in Monrovia, Liberia.; h Division of Global HIV and TB, Centers for Disease Control and Prevention-Zambia, Lusaka, Zambia.; i Infectious Diseases Institute, Makerere University, Kibaale, Uganda.; j Bureau for Global Health, U.S. Agency for International Development, Washington, DC, USA.; k Bureau for Global Health, U.S. Agency for International Development, Washington, DC. Now with ICF, Rockville, MD, USA.

## Abstract

Saving Mothers, Giving Life (SMGL), a 5-year initiative implemented in selected districts in Uganda and Zambia, was designed to reduce deaths related to pregnancy and childbirth by targeting the 3 delays to receiving appropriate care at birth. While originally the “Three Delays” model was designed to focus on curative services that encompass emergency obstetric care, SMGL expanded its application to primary and secondary prevention of obstetric complications. Prevention of the “first delay” focused on addressing factors influencing the decision to seek delivery care at a health facility. Numerous factors can contribute to the first delay, including a lack of birth planning, unfamiliarity with pregnancy danger signs, poor perceptions of facility care, and financial or geographic barriers. SMGL addressed these barriers through community engagement on safe motherhood, public health outreach, community workers who identified pregnant women and encouraged facility delivery, and incentives to deliver in a health facility. SMGL used qualitative and quantitative methods to describe intervention strategies, intervention outcomes, and health impacts. Partner reports, health facility assessments (HFAs), facility and community surveillance, and population-based mortality studies were used to document activities and measure health outcomes in SMGL-supported districts. SMGL's approach led to unprecedented community outreach on safe motherhood issues in SMGL districts. About 3,800 community health care workers in Uganda and 1,558 in Zambia were engaged. HFAs indicated that facility deliveries rose significantly in SMGL districts. In Uganda, the proportion of births that took place in facilities rose from 45.5% to 66.8% (47% increase); similarly, in Zambia SMGL districts, facility deliveries increased from 62.6% to 90.2% (44% increase). In both countries, the proportion of women delivering in facilities equipped to provide emergency obstetric and newborn care also increased (from 28.2% to 41.0% in Uganda and from 26.0% to 29.1% in Zambia). The districts documented declines in the number of maternal deaths due to not accessing facility care during pregnancy, delivery, and the postpartum period in both countries. This reduction played a significant role in the decline of the maternal mortality ratio in SMGL-supported districts in Uganda but not in Zambia. Further work is needed to sustain gains and to eliminate preventable maternal and perinatal deaths.

## Abstract (precis)

The Saving Mothers, Giving Life initiative used 3 coordinated approaches to reduce maternal deaths resulting from a delay in deciding to seek health care, known as the “first delay”: (1) promoting safe motherhood messages and facility delivery using radio, theater, and community engagement; (2) encouraging birth preparedness and increasing demand for facility delivery through community outreach worker visits; and (3) providing clean delivery kits and transportation vouchers to reduce financial barriers for facility delivery. These approaches can be adapted in other low-resource settings to reduce maternal and perinatal mortality.

## INTRODUCTION

### Three Delays That Contribute to Maternal Mortality

Globally, more than 300,000 women die each year due to complications of pregnancy and childbirth, with 99% of these deaths occurring in developing countries.^1^ Approximately 2 million newborns die during their first week of life each year, and an additional 2.6 million are stillborn from complications during pregnancy or delivery.^2^^,^^3^ Effective interventions exist to prevent the majority of these deaths; however, these interventions are often unavailable or inaccessible in many countries in sub-Saharan Africa where the greatest burden lies.^4^^–^^6^

Ending preventable maternal and perinatal deaths while ensuring health and well-being and enabling environments (i.e., survive, thrive, and transform) are the main priorities for the United Nations' Sustainable Development Goals and Global Strategy for Women's, Children's, and Adolescent's Health (2016–2030).^7^^,^^8^ However, equitable access to emergency obstetric and newborn care (EmONC) remains a challenge in many countries, particularly where fertility and mortality levels are high. In low- and middle-income countries, only 1 in 5 pregnant women who experiences pregnancy complications receives EmONC.^9^ Since pregnancy complications are often unpredictable, timely access to quality EmONC is essential to reducing maternal and perinatal deaths.^10^^,^^11^

Although pregnancy complications constitute the diagnosable conditions that lead to maternal deaths, underlying non-medical factors are also important contributors to maternal mortality in developing countries. A large proportion of women die because of (1) delayed recognition of a pregnancy complication and decision to go to a facility, (2) delays in reaching an emergency obstetric care facility, and (3) lack of receipt of timely, adequate, and appropriate obstetric care at a health care facility. Strategies designed to reduce the burden of each of these 3 delays that contribute to maternal deaths can help improve maternal and infant survival. The “Three Delays” model is a useful conceptual and practical framework that can help identify where and when maternal deaths occur and the most appropriate actions on the pathway to preventing future maternal and infant deaths.^12^

The Three Delays model can help identify where and when maternal deaths occur and the most appropriate actions to prevent future maternal and infant deaths.

Originally designed to analyze barriers to EmONC, the 3-Delays model used by the Saving Mothers, Giving Life (SMGL) initiative was applied more broadly to select interventions aimed at reducing barriers to (1) seeking facility-based care during pregnancy, birth, and the postpartum period; (2) reaching facility-based care for routine and complicated births; and (3) receiving timely quality preventive and curative interventions included in facility-based delivery care. Delays in deciding to seek care, including timely recognition of complications (first delay) and in identifying and reaching a health facility (second delay) relate directly to problems with access to care, encompassing factors at the individual, household, community, and health systems level. Contributors to the first and second delays include financial barriers, reluctance to seek care because of demeaning or perceived low quality care, geographic distance from a health care facility, road quality, and lack of transport availability. These factors have been widely recognized as contributing to high levels of maternal and neonatal mortality.^12^^–^^14^ Once a woman has reached a health facility, the delay in receiving adequate and timely care (third delay) relates to factors in the health care facility that are also critical for programs to address. If health facilities cannot provide timely emergency care (i.e., open 24 hours per day/7 days per week, well-staffed, well-equipped, and able to provide an array of lifesaving interventions), addressing the first 2 delays does not improve survival, and in fact may negatively affect perceptions of facility care and demand for health services.

Studies based on maternal death reviews with verbal autopsies differ in their conclusions about which of the 3 delays contributes most to maternal deaths and have found that often a single maternal death may be the result of multiple delays. They also suggest that the relative contribution of the delays may differ according to the study setting and sociocultural, geographic, and health systems context. Although programmatic evaluations in Haiti, Malawi, and Zambia using the Three Delays model suggest that the first and third delays contributed most to preventable maternal and newborn deaths,^14^^–^^16^ other studies have found that the first,^17^ second,^18^ or third delays,^19^ respectively, contribute most to maternal and newborn deaths. Thus, while the predominance of a certain type of delay may differ across health systems and country contexts, assessing and addressing all 3 delays is critical in designing and implementing comprehensive safe motherhood strategies. In addition, factors related to experiencing the 3 delays are often interrelated, overlapping, and complex, with rural, poor, and less educated women often experiencing all 3 delays.^14^

### The Saving Mothers, Giving Life Initiative and the Three Delays Model

The SMGL initiative is an innovative model that brought together diverse public- and private-sector partners in a collaborative effort to dramatically and rapidly reduce the number of maternal and newborn deaths that occur during childbirth and in the period immediately following in selected districts of Uganda and Zambia. Nigeria Cross River State joined SMGL in 2015 (results not included in this analysis).^20^

SMGL simultaneously implemented multiple interventions to target all 3 delays by applying a comprehensive approach to strengthen district health systems. The goal of the SMGL interventions was to ensure that every pregnant woman has access to and uses safe, basic delivery services and, in the event of an obstetric complication, can reach lifesaving EmONC within 2 hours.

Beginning in 2012, SMGL introduced interventions in communities and health facilities (public and private) in 4 pilot “learning” districts each in Uganda and Zambia. SMGL approaches included: (1) generating demand for antenatal, facility delivery, and postpartum care; (2) raising awareness and facilitating action on birth planning, understanding pregnancy danger signs, HIV testing and treatment, family planning services, and postpartum check-ups; (3) upgrading and equipping health care facilities with necessary medical commodities and supplies, including safe blood; (4) hiring, training, and mentoring mid- and high-level staff to increase the number and geographic distribution of quality basic and comprehensive EmONC services with 24 hour coverage; (5) strengthening linkages between communities and facilities through integrated communications and transportation systems and opening of new maternity waiting homes; and (6) increasing capacity of district health systems and personnel to manage and use health management information systems.^21^

For the purposes of reporting the major SMGL intervention strategies, intervention outcomes, and health impacts, we have organized findings according to the Three Delays model in 3 separate articles (this article plus 2 companion articles published in this SMGL supplement). However, it is important to recognize that there is a great deal of overlap among the delays and that the underlying contributors to delayed or inadequate maternal care are often cross-cutting and complex.

### Effective Interventions to Reduce the First Delay

**FIGURE 1:**
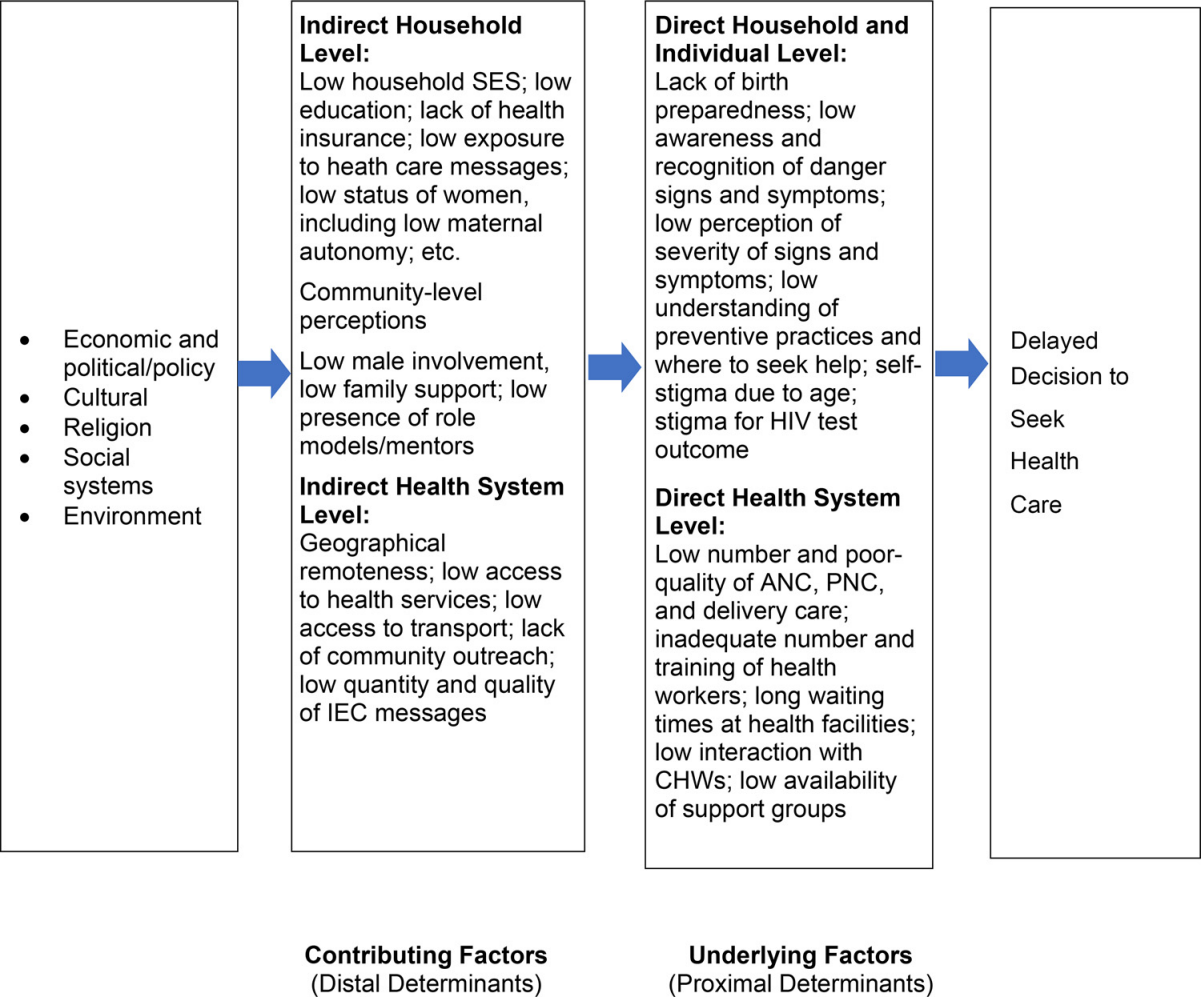
Conceptual Framework to Explain the First Delay in Deciding to Seek Care Abbreviations: ANC, antenatal care; CHW, community health worker; IEC, information, education, and communication messages; PNC, postnatal care; SES, socioeconomic status.

The first delay encompasses numerous barriers that can affect a woman and her family's awareness of a serious complication or timely decision to seek health care. These include broad environmental factors; indirect community, household, and health systems factors; and direct factors related to the household or individual's ability to recognize the need for health care, have a plan in place, and initiate action to reach care, or related to the availability and quality of the health system (Figure 1). Interventions to reduce the first delay address many of these barriers, including individuals' and households' ability to recognize the need for health care, having a birth plan in place, and having adequate financial and logistic resources to access care.

The first delay encompasses numerous barriers that can affect a woman and her family's awareness of a serious complication or timely decision to seek health care.

Literature on barriers to accessing delivery care indicates that individual experiences, financial and decision-making autonomy, and community and societal norms play a major role in women's decisions.^22^^–^^24^ Past negative experiences and perceived poor quality of care at facilities, including the perceptions that facilities are underequipped or that clinic staff are disrespectful, can discourage women and their families from seeking a facility delivery.^25^^–^^27^ Other studies suggest that community and family support are also important drivers of seeking facility care, sometimes superseding negative attitudes about quality of care in the decision to go to a facility.^22^^,^^23^

Community outreach and engagement, through deployment of community health workers (CHWs), can be effective means to increase facility deliveries and use of maternal and child health services.^28^^–^^30^ CHWs can provide linkages between the community and health care facilities along the continuum of care, utilize existing community networks to identify and communicate with pregnant women, engage with local leaders, and promote health messages to increase birth planning, awareness of pregnancy danger signs, and facility delivery.^28^^–^^30^ Facility- and community-level interventions that promote birth preparedness, recognition of complications, and referrals from CHWs to facilities are associated with increased facility-based births.^31^ CHWs can also promote preventive health services. Birth planning and preparedness, as well as identification of underlying maternal risk factors and health conditions, often begin during antenatal care (ANC) visits. ANC provides an ideal opportunity to educate pregnant women about the danger signs of a pregnancy complication and the need to have a birth plan (e.g., saving money, identifying a birth location, arranging and planning for transportation).

Financial and geographic barriers are also important drivers of decisions to seek facility care. Financial barriers that deter seeking facility delivery services are associated with the cost of the delivery itself but also include affordability of transportation to the facility and purchasing medical supplies that must be brought to the health care facility at the time of delivery. Strategies such as voucher incentives and distributing clean delivery kits (CDKs) have been shown to increase facility-based delivery rates.^32^^,^^33^ Finally, accessibility barriers also include geographic distance to a health facility. In Kenya, researchers found that women who live within 2 kilometers of an obstetric facility were more likely to deliver in a facility.^34^

SMGL recognized from the start the critical reality that in Africa women's male partners, extended families, and communities play a crucial role in mothers' health-seeking behaviors. Thus, women in Africa are not always able to make health decisions on their own. The SMGL initiative sought to improve access to safe delivery in health facilities by supporting communities to become more engaged, encouraging families to have a birth plan, providing pregnant women and their partners with information about the danger signs during pregnancy and birth, and addressing social, cultural, and gender barriers to appropriate care. Community outreach activities by community health volunteers (Village Health Teams [VHTs] in Uganda and Safe Motherhood Action Groups [SMAGs] in Zambia) advocated for birth preparedness, promoted health practices, and encouraged ANC visits, facility delivery, and postpartum care. They were also crucial in the distribution of birth plans (Zambia); marketing CDKs containing supplies necessary for birth and newborn care to women who came to deliver in facilities (Uganda and Zambia); and distribution of transport subsidies to increase health care facility use (Uganda).^20^ These activities were supplemented with radio and print media campaigns, community drama groups, and community advocacy through “Mama Ambassadors” in Uganda and “Change Champions” in Zambia.

### National and SMGL-Supported District Contexts in Uganda and Zambia in Relation to the First Delay

At the outset of the SMGL initiative, the 2011 Uganda Demographic and Health Survey (DHS) revealed that almost all (95%) pregnant women in the country received at least some antenatal care, including 48% who attended 4 or more visits.^35^ Nationally, 50% reported having received information during ANC visits on pregnancy danger signs. Overall, 57% of Ugandan women delivered in health facilities, including 52% of women in rural areas who reported a facility delivery. Most women of reproductive age (65%) reported that they have serious problems in accessing health care, including 49% who said that getting money for treatment was a problem and 41% who said that distance to care is an important barrier.

The 2013–2014 DHS in Zambia reported that among women who recently gave birth, almost all (96%) attended ANC, including 56% who attended 4 or more visits during their most recent pregnancy.^36^ The majority of women reported that during ANC they received information about danger signs of pregnancy complications (88%), and that they either discussed a birth plan with a health care provider (91%) or had used a birth plan (88%). Two-thirds (67%) of women delivered in health facilities, but only 56% of rural women reported a facility-based delivery at their last birth. The main reasons for not delivering in health facilities included the facility was too far away or they did not have transportation (32%), followed by labor being unexpected or too short (27%). Two-thirds of women reported receiving postnatal care within 2 days of delivery.

Prior to SMGL, Uganda and Zambia had already taken important steps to identify and implement community health strategies and programs. The Roadmap for Accelerating the Reduction of Maternal and Neonatal Mortality and Morbidity in Uganda (2007–2015)^37^ highlighted the central roles of VHTs and community leaders in ensuring community involvement in health promotion. The roadmap also acknowledged that women's access to preventive health care services can be heavily influenced by husbands or relatives and cultural norms. A woman who has a potential pregnancy complication may not be the one making decisions about her care; rather, societal and familial expectations often take precedence. In addition, because pregnancy and childbirth are seen as normal occurrences, women who deliver without medical assistance may be more highly regarded than those who receive skilled birth assistance.^37^ These cultural factors may contribute to delays in deciding how and when medical care is needed and sought, and highlight the need to conduct community outreach not only for women but also for men and family members. As such, a family and community-centered approach to health promotion is preferable to activities targeting women only.

In Zambia, the Ministry of Health established SMAGs in 2003 to increase the utilization of maternal and newborn health care services.^38^ SMAGs are community-based volunteer groups that aim to deliver essential information on safe motherhood and health prevention practices to men and women. Zambia's Countdown to Millennium Development Goals on maternal and child health prioritized key community health interventions, including expanding the number and scope of SMAGs to be undertaken by 2015 to foster community engagement in safe motherhood.^39^

In both countries, SMGL partners carried out formative research to understand existing district contexts and identify specific factors that influence behaviors before, during, and after delivery. In Uganda, consultative meetings with community, political, religious, and district leaders and postpartum women were conducted to identify barriers to institutional delivery care and best strategies to overcome them. The groups prioritized lack of transportation as a major barrier to timely access to facility-based care, which resulted in the development of a subsidized transport vouchers program. In Zambia, the Communications Support for Health (CSH) project, funded by the United States Agency for International Development (USAID), carried out a qualitative study in selected SMGL districts to better understand the context in which women made care-seeking decisions.^40^ The study found that women knew about antenatal care and the necessity of planning for birth, and they were familiar with pregnancy danger signs; however, only 60% attended 4 or more ANC visits due to difficulty paying for transportation, long wait times, the belief that ANC is only for managing complicated pregnancies, and not seeing the benefit of multiple ANC visits. A 2013 ethnographic study of maternal health-seeking behavior conducted for SMGL in Zambian districts identified cultural beliefs and practices that prevented some women from going to health facilities for care.^41^ The study indicated that some women did not seek antenatal care early in their pregnancy, as is recommended, because they feared bad pregnancy outcomes if they disclosed the pregnancy before it had been announced by an older female relative. The study also found that some women do not deliver in health facilities because they wish to use traditional herbs to promote short labor and reduce bleeding and are not comfortable disclosing this to a health care provider.

Lack of transportation was identified as a major barrier to timely access to facility-based care.

This article examines how the SMGL initiative focused its efforts to address the first delay and integrated its interventions within the district health systems in the learning districts in Uganda and Zambia. We describe the main interventions and approaches within the context of the 3 broad strategies that SMGL implemented to improve care-seeking behaviors:

1. Promote community engagement and empowerment for improved maternal and newborn health.
2. Increase birth preparedness, demand for facility delivery, and use of preventive health care services.
3. Decrease financial and logistic barriers to accessing facility delivery care.

The article also examines process and outcome indicators influenced by community interventions that took place in the SMGL-supported districts including changes in the institutional delivery rate and in antenatal and postpartum care; proportion of health facilities with affiliated CHWs; and maternal mortality due to the first delay.

## METHODS

SMGL used both qualitative and quantitative methods to document intervention strategies, outcomes, and health impacts. To evaluate the impact of the SMGL initiative overall, we compared data collected during the baseline (the 12 months prior to the onset of the initiative; June 2011–May 2012), with data collected during the endline monitoring period (January–December 2016). Programmatic interventions are described as occurring during Phase 1 (June 2012–December 2013) and Phase 2 (January 2014–October 2017) of the SMGL initiative. Further details on the content of the phases is described elsewhere.^20^

### Qualitative Data and Analytic Methods

Qualitative data sources included Phase 1 and Phase 2 project reports and documents submitted by SMGL implementing partners, who collected programmatic data to describe interventions and results. Information on Uganda's inputs came from VHT data and from program reports on Integrated Community Clinic Outreach, community dialogue activities, the “Mama Ambassador” program, “Mama Kit” distribution logs, and radio station invoices and activity reports. Data for Zambia activities were derived from annual performance reports to USAID, Communications Support for Health's final report, and the “Mothers Alive Campaign” Change Champions assessment. Programmatic interventions detailed here generally occurred during Phase 1 and continued into Phase 2. Program data were also derived from reports published by the Columbia University Mailman School of Public Health, which conducted an external evaluation of SMGL at the conclusion of Phase 1.^42^

### Quantitative Data and Analytic Methods

Quantitative sources to assess the results of community-based interventions included data from health facility assessments (HFAs) and the District Health Information System 2 (DHIS2) platforms, as well as population-based data to identify and investigate deaths to women of reproductive age, including those due to maternal causes. Approaches and methods for each of these data sources are fully described elsewhere.^43^ For our study, we compared maternal data collected during the baseline and endline periods.

#### Health Facility Assessments

The SMGL partners implemented HFAs in SMGL-supported learning districts to assess changes in facility infrastructure, functionality, and use.^43^ A total of 105 facilities in Uganda and 110 facilities in Zambia supported throughout the initiative were assessed at baseline and endline. Indicators derived from the HFAs used in this analysis include numbers of deliveries that took place in facilities and the percentage of facilities that reported having affiliated community health outreach workers.

Facility assessments were conducted in virtually all facilities that provide maternity care in SMGL-supported districts. We considered data complete counts rather than a sample and reported indicators as percentages, not subject to sampling error. We calculated the *z* score using the McNemar test for dichotomous responses for matched pairs of data at baseline and endline.

#### District Health Information Systems

In Uganda, SMGL used the Ministry of Health's recently updated DHIS2 to track changes in use of preventive services. The indicators that were used include the proportion of pregnant women in SMGL districts who received 4 or more ANC visits and the proportion of women with at least 1 postpartum visit within 48 hours after delivery in a health facility. Methods for DHIS2 data collection and analysis are described elsewhere.^43^

#### Maternal Mortality Data

To evaluate changes in maternal mortality in SMGL districts, household population data were collected in 2012 and 2017 (through the SMGL Reproductive Age Mortality Study [RAMOS] in Uganda and SMGL District Census in Zambia)^43^ to identify and investigate deaths to women of reproductive age. As part of data collection efforts in both countries, retrospective verbal autopsies were conducted on deaths using the World Health Organization's (WHO's) Maternal Death Surveillance and Response (MDSR) verbal autopsy tool^44^ to identify maternal deaths and their circumstances. Verbal autopsies also provide a better understanding of the social circumstances and decision-making processes preceding a maternal death, and they include qualitative narratives about the pathway from awareness of the onset of a deceased mother's illness or complication to informal or formal treatment received.

Women who experience a first delay may have never attempted to seek health care or may have sought care too late. These 2 groups may differ in their background characteristics, motivations, and decision-making barriers and facilitators. Using verbal autopsies, we examined changes in the proportion of maternal deaths in these 2 groups.

Maternal mortality ratios (MMRs), defined as maternal deaths per 100,000 live births, are based on complete enumeration of deaths identified in communities, so they are not subject to sampling error. The rates are affected by random variation and errors in case detection.^45^ Similarly, percentages were assumed to have some variation or error in measurement. Three different statistical tests were used when comparing the baseline to the endline results. For the mortality ratios, the error was modeled using a Poisson distribution and a *z* score was used to calculate *P* values for significance testing.^46^ For the population percentages, *z* scores based on the normal approximation to the binomial distribution were used to calculate *P* values. The number of maternal deaths and the MMR among women who died of a maternal cause without seeking any health care were also calculated to examine changes in the first delay between baseline and endline.

MMRs and the proportion of deliveries that occurred in facilities rely on the estimated number of live births as the denominator. In Uganda, for both baseline and endline, population statistics were derived from the district-wide SMGL censuses and RAMOS studies, conducted in 2013 and 2017.^43^ In Zambia, at baseline, district-specific population and crude birth rates from the 2010 national census were used to estimate live births for the SMGL-supported districts. At endline, the number of live births was determined by applying district-specific facility delivery rates calculated from the 2017 SMGL census to the district population.^43^ We calculated relative change in indicators by subtracting the baseline value from the endline value and dividing by the baseline.^45^^,^^46^

#### Ethics

The study protocol was reviewed and approved by the U.S. Centers for Disease Control and Prevention (CDC) Human Research Protection Office of the Center for Global Health and by the Ugandan and Zambian Ministries of Health. Written informed consent was obtained for all respondents to the census and verbal autopsy interviews.

## STRATEGIES, INTERVENTIONS, AND RESULTS

### Strategy 1. Promote Community Engagement and Empowerment for Improved Maternal and Newborn Health

Uganda and Zambia SMGL districts employed community-based communication and education strategies to promote safe motherhood messages, increase community awareness of enhanced delivery services in facilities, and engage community leaders and “Change Champions” in promoting the SMGL initiative (Table 1). In Uganda, SMGL used radio programming to broadcast safe motherhood messages from 6 local radio stations about 10 times per day throughout the life of the SMGL initiative (broadcasted 36,146 times during SMGL Phase 1).^42^ It also conducted radio talk shows that included panels of local leaders and technical experts discussing the importance of facility delivery for improving maternal and neonatal health outcomes. In Zambia, radio “spots” emphasized the advantages of facility delivery and encouraged family members to support pregnant women in seeking facility care. Radio messages in Zambia were primarily broadcast during SMGL Phase 1, when approximately 4,000 radio spots were aired.^40^

**TABLE 1.** Saving Mothers, Giving Life Interventions to Reduce the First Delay, 2012–2017

SMGL Strategies and Approaches	Country-Specific Interventions	
	Uganda	Zambia
Strategy 1: Promote community engagement and empowerment for improved maternal and newborn health		
Approach 1.1: Implement community-based communication and education messages on safe motherhood via mass media and community events	Displayed posters with SMGL messages in public places to promote safe motherhood Held talk shows on local radio stations with technical experts and local leaders (political and religious local leaders, local safe motherhood champions) Supported local drama groups to perform skits and traditional songs on safe motherhood, raise awareness of danger signs in pregnancy, and promote facility delivery	Broadcasted targeted radio messages, including spots directed specifically to encourage men to actively support their pregnant partners in seeking care Conducted drama performances to increase knowledge about and demand for delivery services and access to care Created and screened a documentary film “Journey to Becoming a Parent”
Approach 1.2: Build stronger partnerships between communities and facilities	Ensured that all SMGL-supported facilities have VHTs trained in accordance to the national training curriculum Mobilized health facility staff, including district coordinators, to supervise the implementation of activities performed by VHTs	Ensured all SMGL-supported facilities had trained SMAGs Mobilized health facility staff, including district coordinators, to supervise the implementation of activities performed by SMAGs
Approach 1.3: Engage communities in monitoring and evaluation and accountability	Trained VHTs to conduct RAMOS data collection in 2012, 2013, and 2017 Trained VHTs to conduct maternal and perinatal death surveillance in their communities	Ensured that SMAGs reported to health facilities on community events (pregnancies, home births, maternal deaths, and stillbirths)
Strategy 2: Increase birth preparedness, demand for facility delivery, and use of preventive health care services		
Approach 2.1: Assist with community activities aimed to increase: Birth preparedness and knowledge of pregnancy danger signs Use of ANC and PNC services Awareness and use of facility-based delivery services	Trained VHTs in every village to provide health education on birth preparedness and pregnancy danger signs Trained VHTs to encourage women to start ANC early, attend at least 4 ANC visits, deliver in a health facility, and use PNC services Supported VHTs to escort women to deliver in a health facility Trained health facility workers to conduct community dialogue meetings, including meetings that sensitized TBAs about danger signs of obstetric complications, and engaged them in emergency facility referrals	Trained SMAGs to provide health education on birth preparedness and pregnancy danger signs Trained SMAGs to encourage women to start ANC early, attend at least 4 ANC visits, deliver in a health facility, and use postnatal care services Supported SMAGs to escort women to delivery in a health facility
Approach 2.2: Extend the delivery system of preventive services: ANC visits HIV counseling and testing Postpartum home care for mothers and newborns Postpartum family planning	Trained VHTs to perform follow-up postnatal visits for mothers and newborns, identify women and newborns with danger signs, and conduct referrals to health facilities when danger signs are identified Organized clinic community outreach to provide ANC, health education, HIV counseling and testing, immunizations, and male involvement education sessions Selected religious, political, and cultural leaders became champions for promoting utilization of maternal and newborn health services Trained “Mama Ambassadors” to set up community dialogue meetings, give health education talks, distribute health commodities, and provide support to midwives	Trained SMAGs to conduct follow-up postnatal visits for mothers and newborns, identify women and newborns with danger signs, and conduct referrals to health facilities when danger signs are identified Distributed birth plans to help pregnant women plan for social support, transport, nutrition, ANC, and PNC Selected religious, political, and cultural leaders became champions for promoting utilization of maternal and newborn health services Trained community “Change Champions” to promote safe motherhood and HIV prevention practices
Strategy 3: Decrease financial and logistic barriers to accessing facility delivery care		
Approach 3.1: Market and distribute CDKs	VHTs marketed CDKs as part of the promotion of institutional deliveries Facility health workers distributed “Mama Kits” to women who delivered in facilities	SMAGs and nurses in SMGL facilities marketed and distributed “Mama Packs” containing diapers, soap, and baby clothes to women who came to a facility for delivery
Approach 3.2: Market and distribute vouchers to subsidize access to facility delivery care services, ANC, and PNC	VHTs promoted and distributed transport vouchers; health facility workers from private facilities marketed and distributed private vouchers The “Boda for mothers” voucher program to transport women by motorcycle for delivery or obstetric emergencies in 3 districts. During Phase 2, “Boda for mothers” was extended to cover transport for 4 ANC visits and 1 postpartum visit, in addition to transport for delivery care Marie Stopes subsidized vouchers for care in private facilities in all districts (“private vouchers”) (Phase 1 only)	No vouchers or subsidies implemented in Zambia
Approach 3.3: Promote community-based loans to increase utilization of facility delivery care services	Established revolving funds for Village Saving Schemes (Phase 1 only)	Community revolving funds were not implemented in Zambia

Abbreviations: ANC, antenatal care; CDKs, clean delivery kits; PNC, postnatal care; RAMOS, Reproductive Age Mortality Study; SMAGs, Safe Motherhood Action Groups; SMGL, Saving Mothers, Giving Life; TBAs, traditional birth attendants; VHTs, Village Health Teams.

In Uganda and Zambia, SMGL used radio programming to broadcast safe motherhood messages.

Both countries used local theater groups and visual media to conduct community outreach about safe motherhood practices in SMGL districts. In Uganda, SMGL used a community-based drama group during Phase 1 to perform during community dialogue meetings (701 drama skits conducted).^42^ Performances dramatized safe motherhood health messages, which were then discussed during community stakeholder meetings. In Zambia, drama skits were conducted in one district in Phase 1. CSH created a documentary film entitled *Journey to Becoming a Parent* for viewing in SMGL districts.

Although data were not systematically captured to measure the reach of these activities throughout the 5-year SMGL initiative, an external evaluation conducted at the conclusion of SMGL Phase 1 (November 2012–August 2013) by the Columbia University Mailman School of Public Health found that nearly 90% of women delivering at SMGL facilities in Uganda and about 50% in Zambia had heard of SMGL. Respondents in Uganda cited radio as the most common source of information about SMGL (45%), and in Zambia, SMAGs were the most frequently cited source (47%).^42^

SMGL fostered stronger partnerships between communities and health facilities in both countries. Uganda mobilized, expanded, and trained existing VHTs, which represent the most basic level of the national health system. Established in 2000 and affiliated with health facilities, VHTs are community resident volunteers who are trained to provide health education to improve health behaviors and increase the uptake of health services. They are also trained to perform home visits, accompany women to health facilities, and report community health events to the health information system.^47^ Similarly, in Zambia, SMAGs were established in 2003 with donor support and scaled up nationally in 2008. They are tasked to educate communities in health prevention practices (including reduction of HIV transmission) and improve access to maternal and newborn health care services.^38^ Both VHTs and SMAGs operate under the supervision of health personnel in governmental health centers. Previous evaluations conducted in Uganda and Zambia demonstrated an increase in facility-based care in communities where these cadres were functional.^38^^,^^48^

The formation, training, and deployment of VHTs in Uganda and SMAGs in Zambia during Phase 1 involved large-scale mobilization efforts and trainings (both initial and refresher trainings), and were among the most extensively implemented aspects of the initiative.^42^ In Uganda, almost 4,000 VHTs were engaged and trained, covering almost every village in the 4 SMGL districts. Baseline 5-day trainings of up to 40 VHTs and parish coordinators and 2 trainers per training were conducted in mid-2012, followed by 2 one-day long refresher trainings (including one for the 2016 RAMOS that used a census-like questionnaire). Training was conducted using the Ministry of Health training curriculum.^49^ Monthly or quarterly meetings between VHTs and parish coordinators were also used as avenues for refreshing knowledge on any observed gaps. To develop and maintain the VHTs' skills and motivation, SMGL trainers and project staff held approximately 2,400 mentorship meetings over the duration of the initiative. In Zambia, more than 1,500 SMAGs were mobilized and trained during a 5-day training (without refreshers). Trainings used an adaptation of the home-based lifesaving skills curriculum originally developed by the American College of Nurse-Midwives in 1998 to promote safe motherhood outcomes. The curriculum was designed to fit Zambia's national goal of promoting facility-based births for all women. It focused on birth preparedness, complication recognition, and lifesaving interventions that should be initiated while waiting for transport to a health facility in the event that an obstetric complication occurs.

Both countries used a cascading training approach beginning with master trainers from the Ministry of Health. They trained district VHT/SMAG trainers, including project staff, who in turn trained the VHTs/SMAGs in trainings organized at the sub-county level. In Zambia, Peace Corps Volunteers also assisted with SMAG training. Each VHT/SMAG received non-monetary incentives at the onset of the initiative (a bicycle with monthly maintenance allowance, a T-shirt with logo, a pair of gumboots, a bag or backpack, an umbrella, and a raincoat). In Uganda, each VHT also received a phone (on closed user group services with the health facility staff). In Uganda, VHTs received a per-diem during RAMOS data collection activities in 2012, 2013, and 2016.

The SMGL initiative significantly increased the number and expanded the functions of the VHTs and SMAGs in all SMGL-supported districts. In both countries, SMGL capitalized on existing national guidelines for recruiting and training community volunteers. Recruitment of women and men as community health volunteers was done through input from community leaders and neighborhood health committees. Traditional birth attendants (TBAs) were given the opportunity to be trained in becoming VHTs and SMAGs or to become “referral agents” to facility delivery, since both governments have policies in place that actively discourage home delivery. Recognizing that TBA-assisted deliveries were a barrier to facility-based care, the SMGL initiative in Uganda prioritized community sensitization about the dangers of unskilled birth attendance through radio talk shows and skits performed by drama groups. Additionally, the implementing partners used geo-mapping to identify hot spots where community deliveries were predominant that were targeted for community dialogue meetings and site visits. Training curricula in both countries included safe motherhood knowledge and skills, specifically for raising awareness of birth planning, pregnancy danger signs, promoting antenatal care, delivery in a health facility with a skilled provider, and conducting postnatal home visits and essential neonatal care. Volunteers were also trained in reporting to health facilities on community events (home births, maternal and perinatal deaths). Refresher trainings, mentorship, job aids, reporting and reference materials, and transportation means (bicycles) were provided by the implementing partners to support these activities.

**FIGURE 2:**
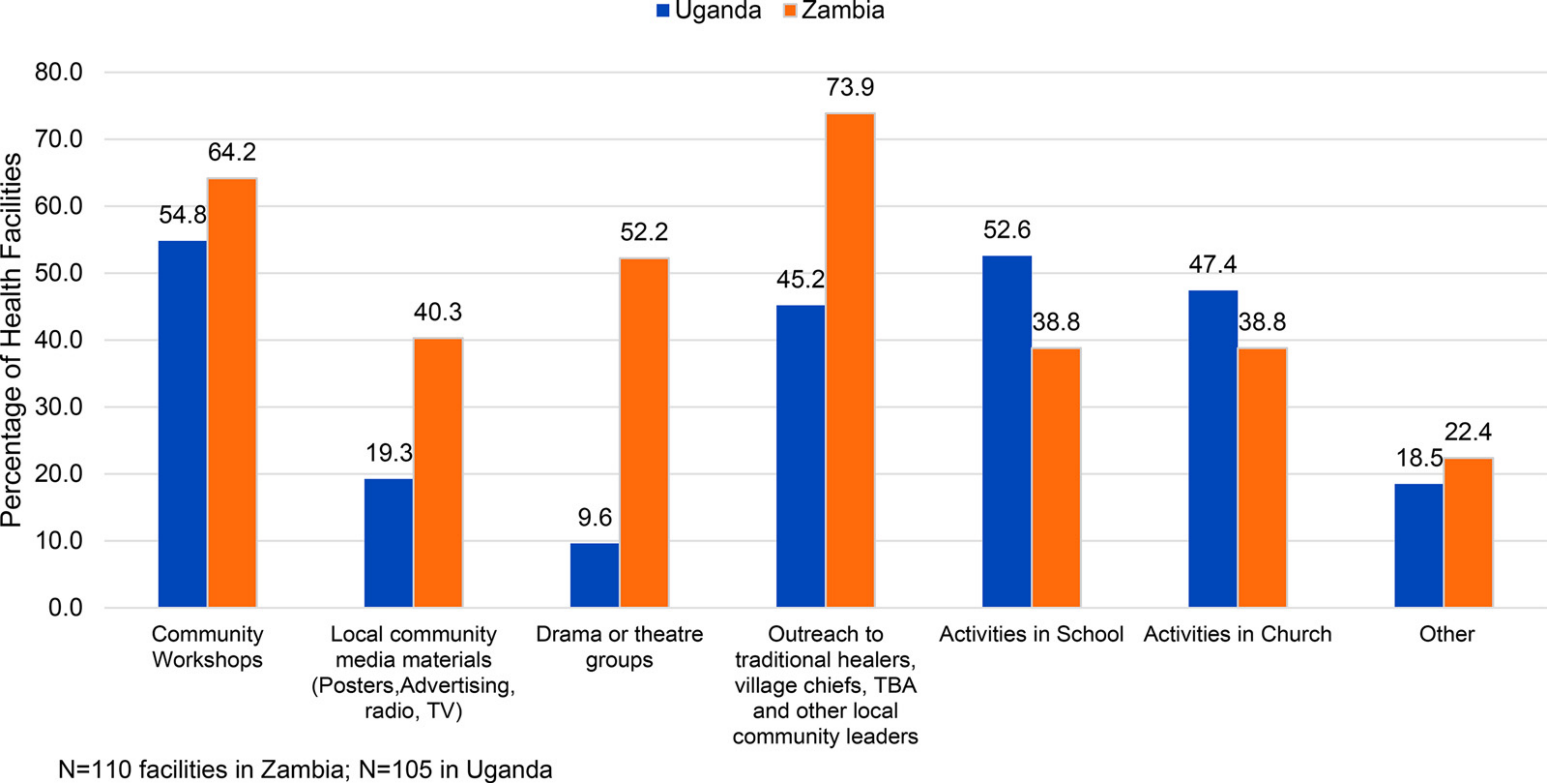
Activities Performed by VHTs/SMAGs in SMGL Districts in Uganda and Zambia, 2016 Abbreviations: SMAGs, Safe Motherhood Action Groups; SMGL, Saving Mothers, Giving Life; TBA, traditional birth attendant; VHTs, Village Health Teams.

The 2016 Uganda HFA documented that the percentage of facilities with affiliated VHTs increased from 18.3% to 91.5% (Table 2). Uganda SMGL facilities with affiliated VHTs reported that the VHTs were engaged in convening community workshops (55%), school- (53%) and church-based (47%) education activities, and conducting outreach to community leaders and TBAs (45%) (Figure 2). Similarly, the 2016 endline HFA in Zambia documented that the percentage of facilities having associated SMAGs increased from 63.8% to 96.3% over the course of SMGL (Table 2). HFA respondents indicated that SMAGs were engaged in outreach activities with community leaders and TBAs (74%), convening community workshops (64%), supporting drama groups (52%), organizing mass media announcements (40%), and conducting school- (39%) or church-based (39%) education activities (Figure 2).

**TABLE 2.** SMGL Outcomes Associated With Strategies to Reduce the First Delay, by Country, 2011–2016

Outcomes	Baseline (Jun 2011–May 2012)	Endline (Jan–Dec 2016)	% Relative Change^a^	Significance Level
Uganda				
Facilities that reported having an associated VHT (%)^b^	18.3	91.5	+400	***
Institutional delivery rate, all facilities (%)^b^	45.5	66.8	+47	***
Institutional delivery rate, EmONC facilities (%)^b^	28.2	41.0	+45	***
Institutional delivery rate, non-EmONC facilities (%)^b^	17.3	25.8	+49	***
Pregnant women who had 4 or more ANC visits (%)^c^	46.1	56.7	+23	***
Women who had a postpartum care visit within 48 hours (%)^c^^,^^d^	15.3	17.7	+16	***
Zambia				
Facilities that reported having an associated SMAG (%)^b^	63.8	96.3	+51	***
Institutional delivery rate, all facilities (%)^b^	62.6	90.2	+44	***
Institutional delivery rate, EmONC facilities (%)^b^	26.0	29.1	+12	***
Institutional delivery rate, non-EmONC facilities (%)^b^	36.7	61.1	+67	***

Abbreviations: ANC, antenatal care; DHIS2, District Health Information System 2; EmONC, emergency obstetric and newborn care; HFA, health facility assessment; PNC, postnatal care; SMAG, Safe Motherhood Action Group; SMGL, Saving Mothers, Giving Life; VHT, Village Health Team.

*** *P*<.01.

a Percentage change calculations are based on unrounded numbers.

b HFA data (Uganda N=105 facilities; Zambia N=110 facilities).

c DHIS2 data, using estimated live births as denominator.

d Baseline data include PNC visits beyond the first 48 hours, so the percentage increase is conservative.

In Uganda, VHTs are actively engaged in the health management and information system and submit monthly reports on selected community health events.^47^ The SMGL partners and the CDC Division of Reproductive Health built on this platform to create a comprehensive maternal and neonatal death surveillance and response system at the village level. They trained and monitored approximately 3,800 VHTs to identify deaths among women of reproductive age and report them to sub-district health coordinators monthly. Households with deaths among women of reproductive age were visited by a trained verbal autopsy team. If the death occurred during pregnancy or delivery or within 2 months of a delivery, the team collected information about the circumstances of death and contributing factors, using the verbal autopsy tool.^44^ Beginning in 2015, Uganda VHTs supported the integration of neonatal deaths surveillance into the MDSR system, following procedures recommended by WHO.^51^

### Strategy 2. Increase Birth Preparedness, Demand for Facility Delivery, and Use of Preventive Health Care Services

SMGL partners engaged existing cadres of community public health workers to conduct outreach to SMGL districts and communities to encourage birth preparedness and knowledge of pregnancy danger signs, encourage use of ANC and postnatal care services, and increase awareness and use of facility delivery services. In both Uganda and Zambia, cultural norms place importance on the role of the woman's partner, family, and even community in making health decisions during pregnancy and childbirth. SMGL sought to address gender, social, and cultural barriers to facility-based care by encouraging families and communities to recognize the importance of having a birth plan, attending ANC, and delivering in a health care facility.

VHTs and SMAGs delivered an array of interventions aimed at women's education on birth preparedness and referral to health facilities for skilled maternal and newborn health services. In both countries, these community volunteers identified pregnant women in their communities, informed them about birth planning and pregnancy danger signs, promoted ANC visits, and often accompanied women for delivery in a health facility. A minimum of 4 ANC visits were heavily promoted in order to get women connected early in pregnancy to a health care facility, increase the identification of high-risk pregnancies, and encourage facility deliveries. In Zambia, SMAGs distributed birth plan documents to remind pregnant women of the steps they need to take to have a healthy pregnancy and delivery, including information about nutrition, ANC, pregnancy danger signs, birth planning, and postnatal care. Stocks of birth plans were distributed at clinics in SMGL-supported districts, with approximately 400,000 provided over the course of the project. SMAGs distributed these birth plans continuously, with approximately 70% of pregnant women receiving and using the birth plan.^52^

Community outreach workers in both Uganda and Zambia delivered an array of interventions to educate women about birth preparedness and encourage delivery in a health facility.

VHTs and SMAGs were also trained to perform follow-up postnatal home visits for mothers and newborns, identify mothers and newborns with danger signs, and conduct referrals to health facilities when danger signs were identified. In Uganda, the work performed by community volunteers was aided by health facility workers, who periodically organized community dialogue meetings, including meetings with community TBAs to sensitize them about danger signs of obstetric complications and engage them in facility referrals.

The SMGL initiative also placed emphasis on postpartum family planning and increased identification and treatment of pregnant women and newborns with HIV infection to prevent mother-to-child transmission of HIV/AIDS. VHTs, SMAGs, community champions, and SMGL health care facility workers included promotion of these topics in their community outreach and education activities. In Uganda, health facility workers provided community outreach services related to blood pressure screening and other focused ANC services, health education, HIV counseling, testing and referrals, immunizations, and male involvement education sessions.

In both countries, the implementing partners engaged traditional and local government leaders, as well as religious leaders, to increase community engagement and access to maternal and newborn health services. They partnered with influential community members to assess the needs of their communities; identify local priorities, opportunities, and challenges; and develop approaches for recruitment, training, and retention of volunteers. Further, community leaders were actively engaged in community dialogues, health promotion activities, and the facilitation of volunteers' work (recruitment, motivation, oversight, and accountability).

The SMGL initiative promoted women champions to talk about ways in which mothers should address their own health and their children's health. This has been proven a successful health promotion strategy that empowers women at the same time.^28^ In Uganda, SMGL recruited and trained a cadre of “Mama Ambassadors,” women community leaders who reinforced maternal health messages at community dialogue meetings, led clinic outreach events, provided health education to mothers at ANC visits in health facilities, and participated in radio talk shows. This cadre also provided non-technical support in antenatal and postpartum care to midwives during busy clinic days. During Phase 1, 78 women served as Mama Ambassadors, and the number increased slightly to 87 during Phase 2. In Zambia, SMGL trained and deployed 350 community leaders to be “Mothers Alive Campaign Change Champions.” Change Champions were often traditional leaders in chiefdoms tasked with tracking and reporting maternal deaths and promoting safe motherhood. Change Champion leaders identified and addressed specific challenges to meet their community needs, such as soliciting and receiving an ambulance from the Ministry of Health, initiating a garden and food safety net to improve maternal nutrition, soliciting local business contributions to build a mother's waiting shelter, and constructing a new rural health center.

HFA and pregnancy outcome monitoring data indicate that the promotion of maternal and newborn health services was effective; facility deliveries rose significantly in SMGL districts in both Uganda and Zambia during Phase 1, and the increased levels were maintained or continued to increase over the course of Phase 2.^51^ In Uganda, the proportion of all births that took place in facilities rose from 45.5% to 66.8% (47% increase) over the 5-year SMGL initiative (Table 2). Increases in facility deliveries occurred both in facilities that were equipped to perform a full range of EmONC functions (45% increase) and in facilities that provided delivery services but were not categorized as EmONC facilities (49% increase). Likewise, in Zambia facility deliveries in the SMGL-supported districts increased from 62.6% to 90.2% (a 44% increase), with a 12% increase in deliveries in EmONC facilities and a 66% increase in non-EmONC facilities.

Facility deliveries rose significantly in SMGL districts in both Uganda and Zambia during the first phase of the initiative, and the increased levels were either maintained or continued to increase during the second phase.

Between baseline and endline assessments of the SMGL initiative in Uganda, the proportion of pregnant women in SMGL districts who had 4 or more ANC visits increased by 23% (from 46.1% to 56.7% of pregnant women) (Table 2). A comparison of baseline and end of Phase 1 national DHIS2 data determined that the proportion of women with 4 or more ANC visits was consistently higher in SMGL districts than in neighboring districts in Western Uganda.^53^ The proportion of Ugandan women with a postpartum care visit within 48 hours of delivery, though much lower, also increased significantly during SMGL implementation (from 15.3% to 17.7%). Comparable data were not available for Zambia.

### Strategy 3: Decrease Financial and Logistic Barriers to Accessing Facility Delivery Care

Women and families' reasons for not seeking facility care or for delaying the decision to go to a facility also include financial barriers. In both Uganda and Zambia, the SMGL initiative distributed CDKs at facilities to provide incentives for facility delivery. To encourage women to deliver in facilities and to facilitate sanitary births, SMGL Uganda distributed “Mama Kits” to pregnant women who came to a facility. Mama Kits contained items that women are often required to purchase and bring with them to a facility delivery, including a plastic sheet, gauze, razors, syringes, disposable gloves, eye ointment, and soap. Each kit also included baby sheets, a baby shawl, and a child growth card. SMGL Uganda provided Mama Kits to 15,655 women in the 4 learning districts during Phase 1. Similar “Mama Packs” were made available in 2 SMGL districts in Zambia at health facilities to women who came to deliver.^42^ During Phase 1, about 2,000 Mama Packs were distributed in the 2 districts, but due to concerns about sustainability Mama Packs were discontinued during Phase 2. The Columbia University evaluation of Phase 1 found that, in exit interviews with women who had delivered in a facility, the kits allowed families to save money that could help pay for other necessities, such as transportation.^42^ The evaluation also found the kits were popular, with 25% of the women who participated in an exit interview in Uganda SMGL districts reporting having used the kit for their recent delivery.^42^

SMGL distributed clean delivery kits with items that women are often required to purchase and bring with them to a facility delivery, such as a plastic sheet, gauze, disposable gloves, and soap.

In Uganda, physical and economic accessibility were enhanced through a voucher system that provided access to motorcycles (“boda for mothers”) and subsidized the cost of transportation to delivery services. In 3 Uganda districts where Baylor College of Medicine implemented the SMGL initiative (Kabarole, Kamwenge, and Kyenjojo), transport vouchers substantially enhanced women's access to facility-based births during Phase 1. In addition, vouchers for transport to and use of services in nongovernmental facilities offering childbirth care (including cesarean deliveries) were subsidized and rapidly scaled up by Marie Stopes International during Phase 1. Beginning in 2012, pregnant women were able to buy both vouchers at a minimal cost during ANC or directly from VHTs in their communities. Altogether, the percentage of voucher-supported deliveries in the Baylor implementation districts increased from 15% in April 2012 to 79% 12 months later. Use of boda-for-mothers vouchers increased dramatically (from 3% to 47%), and use of vouchers for accessing and receiving delivery care in nongovernmental facilities almost tripled (from 12% to 32%). During SMGL Phase 2, voucher supply was inconsistent due, in part, to the discontinuation of the vouchers for nongovernmental facilities. However, boda-for-mothers vouchers were expanded during Phase 2 to provide transport not only for reaching delivery care in facilities but also for 4 ANC visits and 1 postnatal care visit. In 2016, nearly 1 out of 4 women who delivered in any health facility in the 3 Ugandan districts used transport vouchers to reach delivery care.

Baylor Uganda complemented the voucher program with the provision of small community grants given to start community-based revolving funds. However, it was not clearly documented how many users benefited from such loans, whether the initial grants generated substantial community contributions, and which members of the community were expected to contribute.

### Maternal Mortality in SMGL-Supported Districts

Over the 5 years of SMGL implementation, the district-wide MMR in Uganda declined from 452 to 255 maternal deaths per 100,000 live births, and in Zambia, from 480 to 284 maternal deaths per 100,000 live births.^43^

Over the 5-year initiative, the district-wide MMR in Uganda declined from 452 to 255 maternal deaths per 100,000 live births; about half of this decline was associated with increased care seeking for obstetric complications.

In Uganda SMGL-supported districts, 342 women died of a maternal cause between June 2012 and May 2013, compared with 222 women between January and December 2016.^41^ At baseline, care-seeking information collected through verbal autopsies was available for 322 women who died of a maternal cause; of these, 86 women (26.7%) did not seek any care outside the home—including care from a health facility or from a TBA, a traditional healer, or a pharmacist/drug seller. At endline, of 222 women who died of a maternal cause, only 21 (9.5%) did not seek any care outside the home. Applying the baseline proportion of maternal deaths for which no care was sought (26.7%) to the observed endline number of 222 maternal deaths, we would have expected 59 deaths to women who did not seek care to have occurred during the endline period under baseline care-seeking patterns. Since only 21 women died without seeking care at the endline, we infer that 38 deaths were averted through interventions that increased care seeking outside the home. This number of deaths averted accounts for a 23% decline in the overall MMR (from 452 to 349 deaths per 100,000), or about half of the overall 44% MMR decline.

Among women who died of a maternal cause who sought or attempted to seek any care outside the home, the median duration of the delay from the onset of complications to seeking any health care was 5 hours at baseline and 3 hours at endline (data not shown). Almost 4 times more women who died had sought or attempted to seek care within the first hour of symptom onset at endline compared with baseline (26.9% vs. 7.2%, respectively).

In Zambia SMGL districts, 200 women died of a maternal cause during the 12 months preceding the baseline census, compared with 135 during the 12 months preceding the endline census. Of those, 42 women (21%) who died of a maternal cause did not seek any care outside the home at baseline, compared with 30 (22%) at endline. Applying the baseline proportion of 21% of maternal deaths for which women did not seek care to the number of deaths at endline (135), we would have expected 29 maternal deaths to have occurred if baseline care seeking had not changed. Since this is similar to the documented number of 30 women who died without seeking care, we infer that the increase in seeking care was not a substantial contributor to the MMR decline in Zambia.

At baseline, among the 158 women who died of a maternal cause despite the fact they sought or attempted to seek care prior to their death, the median delay to seek care was 24 hours. At endline, the same median duration of delay in seeking care was reported for the 105 mothers who died after they sought or attempted to seek care. The proportion who sought any care within the first hour from onset of symptoms also changed little (21.1% at baseline and 17.7% at endline) (data not shown).

Verbal autopsy narratives illustrate the circumstances and barriers encountered in seeking care among women who died of maternal causes. In the case vignette in the Box, Hellen's mother-in-law describes the factors that affected Hellen's decision not to seek facility delivery. Her delay in seeking care was influenced by past personal experiences and wanting to avoid a third cesarean delivery, advice from a friend and a religious leader, and other sociocultural factors. At each step of her decision-making process, these factors delayed Hellen and her family's recognition of the seriousness of her condition and added more barriers to receiving emergency health care interventions that could have saved Hellen's life. A better understanding of her high-risk status associated with prior cesarean deliveries could have been part of a birth plan for Hellen.

BOX **Verbal Autopsy Case Example of the First Delay: Hellen's Story**

Hellen, a 24-year-old woman from Uganda, died giving birth to her third child after 2 previous cesarean deliveries. The transcript below comes from a verbal autopsy interview with her mother-in-law. Details have been added in brackets to clarify meaning; names of persons, places, and dates have been changed to protect confidentiality.

Hellen felt labor pains but kept quiet and did not tell anyone for 2 days. After 2 days, the labor pains disappeared at night, but Hellen asked her husband not to tell anyone about it because she was afraid to go to the hospital as she thought they would operate on her again. She left the house and went to talk to a friend who had earlier advised her that it was possible for her to push [deliver] the baby on her own and had taken her to the traditional birth attendant. The traditional birth attendant had assured her that she could deliver at home even though her last 2 births had been by cesarean delivery. Hellen and her friend went to the pastor who prayed and gave Hellen herbs to take to aid the delivery. Again, Hellen was assured by the pastor and her friend that she could push [deliver] the baby. Hellen went back to her own house but started bleeding. She then called her mother and told her that she was becoming weak. Hellen's mother called a vehicle to take her to the hospital. As soon as the vehicle came, Hellen was carried to it, but before they could even leave, they realized she was dead.

## DISCUSSION

The SMGL approaches to addressing the first delay were predicated on the assumption that increased utilization of maternal and newborn health services and improved health outcomes cannot be achieved without community engagement and empowerment. The SMGL initiative focused heavily on allocating resources to promote community engagement, increase birth preparedness, educate communities about the benefits of facility delivery, increase supply of and demand for newly expanded facility resources, and reduce barriers to accessing health services.

The initiative recognized that engaging community members as active participants in addressing their own communities' health issues is critical. Activities were designed to raise individual and community awareness on safe motherhood and the benefits of facility delivery, build partnerships between communities and health facilities, and deliver health education and selected preventive services outside health facilities through community health volunteers, community champions, and outreach clinics. In Uganda, engaging communities and community volunteers in the process of identifying and assessing causes of maternal and newborn deaths and in measuring changes in mortality over time helped government efforts to promote accountability in accordance with their global commitments. SMGL interventions also sought to reduce financial barriers to facility care with the distribution of CDKs in both countries and voucher systems in Uganda. Dramatic increases in facility deliveries in SMGL districts, as well as use of CDKs and vouchers, provide evidence that these strategies were likely effective in promoting greater awareness of and access to facility care.

Engaging community members as active participants in addressing their own communities' health issues is critical.

### SMGL's Successes

The barriers addressed by SMGL in communities covered major known contributors to not seeking facility care, including lack of knowledge of the danger signs of pregnancy complications, mistrust or poor perception of facility care, and lack of material resources for transportation or birth supplies. During Phase 1, the Columbia University external evaluation reported that improved facility care in SMGL-supported districts fostered greater community recognition of the value of and need for receiving maternity care in facilities and increased the likelihood that women would seek facility delivery. As women's confidence and trust in providers and in the quality of health services grew, they began returning to the facilities with their children for general maternal and child health services and for future births.^40^

Implementing partners conducted community outreach on safe motherhood issues in SMGL-supported districts. They engaged with and expanded existing Ministry of Health community cadres—Village Health Teams (Uganda), Safe Motherhood Action Groups (Zambia), newly trained women champions (Mama Ambassadors in Uganda and community Change Champions in Zambia)—and mobilized health facility workers to promote and support community activities. VHTs and SMAGs formed the backbone of SMGL's community engagement efforts by raising community awareness of safe motherhood, distributing birth plans and vouchers, escorting women to facilities or maternity waiting homes, performing home visits, and collecting and reporting data for the initiative. These community cadres became trusted sources of information, respected for their dedication to and passion about preventive practices, birth companionship to delivery care, and postpartum home visits. In Uganda, monthly reports from VHTs strengthened monitoring and evaluation of SMGL efforts and laid the foundation of a national model for maternal and neonatal death community surveillance.

Implementing partners utilized multiple forms of communication to reach the community with messages about safe motherhood and the benefits of facility delivery. By using many avenues (face-to-face visits by VHTs and SMAGs, radio programs, community meetings, drama groups, health education by health care providers), SMGL ensured broad segments of the community, including pregnant women, their families, men, and elders, received information. SMGL's approach of extending the information, education, and communication activities to the whole community increased the possibility of shifting community norms to promote long-term change in attitudes and behaviors that support facility-based pregnancy and delivery care.

Financial incentives, through CDKs (in both countries) and transport and service vouchers (in Uganda), provided women and their families with tangible ways to overcome monetary barriers to accessing facility care. The voucher program in Uganda reduced the impediments of distance to care and the cost of transportation. The Mama Kits and Mama Packs provided women with some of the supplies needed during facility delivery. These strategies were generally popular in the SMGL districts that supported them. Subsidies, incentives, and community health worker outreach supported by SMGL were identified as “active ingredients” of the SMGL initiative at the conclusion of Phase 1.^40^

Both countries demonstrated clear commitments to improve health and well-being by strengthening community health systems, as reflected in the national policies and domestic funding issued prior to the SMGL initiative. In collaboration with national and district stakeholders, SMGL implemented evidenced-based strategies^28^^–^^30^ that were country-defined and driven, extensive, and adequately funded. Although not all activities are financially sustainable without continued donor assistance, the SMGL accomplishments demonstrate that countries can rapidly promote and expand access to health at the community level with additional funding. These successes could also inform identification of community health priorities within the national strategy, as the new Uganda community health roadmap suggests.^55^

The accomplishments of the initiative in relation to health outcomes were documented through extensive monitoring and evaluation activities, including population-based measurement of maternal mortality. Data yielded from these efforts indicate that the SMGL-supported districts experienced significant increases in facility deliveries and declines in maternal mortality. Moreover, care-seeking behaviors among Ugandan women who died of maternal causes improved substantially and the median time between the onset of women's symptoms and the decision to seek care declined, even though these women ultimately did not survive. We estimated that the reduction in the number of maternal deaths among women who did not seek care contributed to about half of the overall MMR decline in Uganda. In Zambia, where institutional delivery rates were high at the outset of SMGL and fewer women did not seek care prior to death, the impact of changes in care-seeking behaviors was negligible.

Although SMGL did not use a comparison group, the independent evaluation in Uganda and Zambia at the conclusion of Phase 1 and a separate study in Kalomo district in Zambia showed greater community awareness, demand for facility-based delivery care, and satisfaction with the services received in SMGL districts when compared with other districts nearby.^40^^,^^53^

### Limitations of the SMGL Approach and Monitoring and Evaluation Methods

Despite SMGL's success in increasing facility deliveries and reducing maternal deaths, the initiative faced notable challenges. Large investments in education messages via mass media and community events could not be carried out beyond Phase 1. Rapid expansion of the activities performed by community health volunteers may not be sustainable, though it is aligned with government priorities.^54^ CDK incentives and transport subsidies for facility delivery were periodically depleted, according to the Phase 1 external evaluation.^40^ Funding delays and changes in implementing partner contracts occurred periodically over the life of the SMGL initiative, causing resource depletion or temporary interruption of community outreach activities.

Other limitations stemmed from the increased demand for SMGL facility services outpacing the supply. In certain areas, SMGL's work to encourage facility delivery led to a rapid increase in numbers of women seeking services that exceeded the facilities' capacity, despite intense efforts to improve and expand facilities and staffing. This sometimes led to facility congestion and overworked health care providers, as well as to the increased possibility that the quality of facility care could be compromised.

In some areas, increased demand for facility services exceeded facilities' capacity.

SMGL was launched rapidly in separate countries and districts and relied heavily on the organizational structure and capacity of different implementing partners to mobilize quickly. This proved challenging for the coordination, intensity, and continuity of SMGL intervention and evaluation approaches across districts and countries. Although general strategies and approaches were shared across the initiative, as shown in Table 1, specific approaches varied according to location. In some instances, approaches were unique to an implementing partner and district context, as is the case of the voucher system implemented in 3 districts in Uganda.

Different intervention approaches and varied resources across implementing partners, districts, and countries, as well as the lack of process evaluations of specific community-based interventions, resulted in an inability to attribute specific community-based messaging or interventions to the successful increases in facility deliveries and improved health outcomes. Although all implementing partners collected data on their level of efforts related to community health activities, they did not use a set of unified indicators nor did they collect these data continuously. Community-based data that may have explained the strength of association between community engagement and improved health outcomes were not collected at endline and hence were not included in the final evaluation. Only the Phase 1 evaluation using exit interviews and focus groups^40^ captured important information on community perceptions, women's attitudes about SMGL services, and use of transport arranged through community mobilization and transport vouchers; comparable data were not collected during Phase 2.

Although extensive monitoring and evaluation activities were implemented for SMGL, these methods focused heavily on measuring effects on health outcomes and much less on process documentation of various programmatic approaches. When process indicators were monitored, they mostly documented Strategies 2 and 3 aimed at increasing facility delivery and use of preventive health services. Systematic data were not collected to directly link inputs and processes of SMGL communications strategies (Strategy 1), community birth planning activities (Strategy 2), or financial incentives (Strategy 3) with health outcomes.

The evidence of SMGL successes in reducing maternal mortality at a higher pace than the rest of the country is strong.^41^ Documenting the role of reducing the first delay in maternal mortality is challenging in the absence of a comparison group that would allow examination of whether there were significant socio-demographic, medical, or other delay-related differences between deceased women and women with obstetric complications who survived. However, verbal autopsy studies often have no comparison group and the effect of SMGL interventions should have been accessible to all pregnant and postpartum women. Since the verbal autopsy respondents were the main caregivers of the deceased women, it is possible that the information about delay in seeking health care may have been affected by personal biases, poor recall of events, or lack of precise reporting of symptoms or timing. Further, the decision to seek care stems from an awareness about the severity of the mother's condition and that health care was needed. Verbal autopsy questions on awareness and decision making may have been interpreted differently by caregivers of women with or without evident obstetric complications prior to deciding to seek care outside the home.

There is strong evidence that maternal mortality in SMGL-supported districts dropped at a higher pace than the rest of the country.

### Addressing the First Delay Within the Context of a Systems Approach

Reducing and ultimately eliminating barriers that contribute to the first delay in accessing health care services is critical to achieve continued reductions in preventable maternal and neonatal mortality. Individual and community engagement aided by political support, program integration, and partnerships are critical drivers of change to improve survival, promote health and well-being, and ensure enabling environments.^7^^,^^8^ The SMGL experience provided valuable lessons and insights into how increased community engagement combined with health systems strengthening within the context of existing national policies and in partnership with national, district, and local stakeholders can be instrumental in achieving mortality decline.

In collaboration with the Ministries of Health in Uganda and Zambia, SMGL implemented a broad array of community health interventions (covering over 90% of communities in the learning districts), that were context-specific, coordinated, integrated along the continuum of care, and aligned with country-defined priorities. SMGL strategies coalesced national efforts to define a comprehensive community health agenda (as illustrated by the new community health roadmap in Uganda) with district-driven priorities both centered on increased community ownership and engagement.

SMGL community health strategies and activities helped stimulate demand for facility delivery care. Facility delivery rates increased, including in those facilities able to provide the complete range of lifesaving interventions that constitute EmONC. Although maternal deaths associated with the first delay declined in the learning districts, recognizing a serious complication and making a timely decision to seek health care in a facility is only the first step of the journey to a safe facility delivery. Deaths associated with the second or third delays remain a serious threat if women have waited too long to seek care, face insurmountable barriers getting to a facility, or receive inadequate care once they arrive at a health care facility. Thus, SMGL's systems approach to addressing all 3 delays is critical, so that programs designed to increase demand for facility maternity care are also able to ensure readily available transport to a facility and an adequate supply of quality facility care. Sustainability for maternal mortality reduction initiatives include building a robust community health system within which community members are aware of and actively engaged in their health care and ensuring that the supply of high-quality care can meet increased demand.

## Notes

**Peer Reviewed**

**Funding:** Saving Mothers, Giving Life implementation was primarily funded by the Office of the Global AIDS Coordinator, the U.S. Agency for International Development (USAID), Washington, DC, the Centers for Disease Control and Prevention (CDC), Atlanta, Georgia, Merck for Mothers, and Every Mother Counts. The funding agencies had no influence or control over the content of this article.
